# Save Us from Our Friends

**Published:** 1891-11-28

**Authors:** 


### Passing Topics.

### "SAVE US FROM OUR FRIENDS."

We do not know what that oft appealed to and occasionally maligned authority—the intelligent foreigner—might have to say on the subject, but to us it appears that a painfully ludicrous aspect has been given to all that appertains to lifeboats by the proceedings of the noisy and self constituted partisans of the several crews who have been recently called upon for service.

To read all the superlative writing for which space has been found day by day in our newspapers, one would be led to suppose that pluck and endurance were so rare among Englishmen that a tale of deed and daring needed more repetition that a nursery story. Added to this so much disposition has been manifested to pit the performances of one boat against another, and to disparage, or at least to damn with faint praise, the doings of all other crews save our own, that it will be remarkable, indeed, if under such tuition good-natured and honourable emulation between the boats does not degenerate in the near future into mean and unfriendly rivalry.

One can forgive a certain contemporary journal an occasional perfervid leader, for "'tis its nature to," and moreover, a general and outspoken acknowledgment of bold deeds is only right and encouraging, but what we do object to is that hysterical and meddlesome people should be allowed too ample opportunities for instituting odious comparisons and magnifying the heroic doings of their favourites at the expense of all other heroes whatever. Really it would almost seem as if some of these wordy roysterers had brought themselves to believe that the tempest and all its terrible accompaniments had been prepared with the sole object of ministering to their epic powers, and we can well imagine the astonishment some of our gallant fellows must feel upon perusing these curious, and, whatever the intention, not always complimentary compositions. Can anything, for instance, be more grotesque than the letter written by a local clergyman to protest gravely that a particular volunteer who took his seat in the Sandgate lifeboat when she gallantly rescued the crew of the *Benvenue*, does not live in Folkestone, as had been most outrageously reported, but in "Cheriton, a large village bordering on the landward of Folkestone." "When," continues the aggrieved scribe, "we see him assigned to Folkestone we feel like the man whose one ewe lamb was stolen from him." Well may the paper which gives insertion to the letter, head it with the words: "Hard on Cheriton," that "large village," which, if we are to accept the testimony of the reverend gentleman, contains but one brave man!

The theme does not gain by the many other communications anent lifeboats. Here we are told of a crew striking for higher wages, and then of one charged with neglect of duty amounting to cowardice, while like policemen, lifeboats are often absent when most wanted. Altogether, the great storm which bade fair to bring about quite a "Lifeboat Boom," has turned out somewhat of a failure in that respect, but it will have accomplished real good if it sets us thinking whether we are altogether right in leaving the great task of rescuing the lives endangered upon our storm-beaten coasts to an unaided voluntary organisation whose efforts command our gratitude, but which lacks needful resources and is necessarily deficient in power to enforce discipline. Agricola.

